# TaiChi and Qigong for Depressive Symptoms in Patients with Chronic Heart Failure: A Systematic Review with Meta-Analysis

**DOI:** 10.1155/2021/5585239

**Published:** 2021-06-24

**Authors:** Wei Jiang, Shaojun Liao, Xiankun Chen, Cecilia Stålsby Lundborg, Gaetano Marrone, Zehuai Wen, Weihui Lu

**Affiliations:** ^1^Department of Cardiology, Guangdong Provincial Hospital of Chinese Medicine, Guangzhou 510120, China; ^2^The Second Affiliated Hospital of Guangzhou University of Chinese Medicine, Guangzhou 510405, China; ^3^Second Clinical Medical College, Guangzhou University of Chinese Medicine, Guangzhou 510405, China; ^4^Health Systems and Policy, Department of Global Public Health, Karolinska Institutet, Stockholm 17177, Sweden; ^5^Key Unit of Methodology in Clinical Research, Guangdong Provincial Hospital of Chinese Medicine, Guangzhou 510120, China; ^6^National Centre for Design Measurement and Evaluation in Clinical Research, Guangzhou University of Chinese Medicine, Guangzhou 510405, China

## Abstract

**Background:**

Depression is a debilitating comorbidity of heart failure (HF) that needs assessment and management. Along with mind-body exercise to deal with HF with depression, the use of TaiChi and/or Qigong practices (TQPs) has increased. Therefore, this systematic review assesses the effects of TQPs on depression among patients with HF.

**Methods:**

Randomized controlled trials (RCTs) that examined the effect of TQPs on depression in patients with HF were searched by five databases (PubMed, Cochrane Central Register of Controlled Trials (CENTRAL), EMBASE, CINAHL, and China National Knowledge Infrastructure (CNKI)). With standardized mean difference (SMD) and 95% confidence intervals (95% CI), random-effects meta-analyses of the effect of TQPs on depressive symptoms were performed.

**Results:**

Of eight included RCTs, seven (481 patients) provided data for the meta-analysis. The pooling revealed that TQPs contribute to depression remission in HF (SMD −0.66; 95% CI −0.98 to −0.33, *P* < 0.0001; *I*^2^ = 64%). Its antidepressive effect was not influenced by intervention duration or exercise setting, but rather by ejection fraction subtype, depressive severity, and depression instruments. The beneficial effects were preserved when the study with the largest effect was removed.

**Conclusion:**

This study suggests that TQPs might be a good strategy for alleviating depressive symptoms in patients with HF. And rigorous-design RCTs, which focus on the identified research gaps, are needed to further establish the therapeutic effects of TQPs for depression in HF.

## 1. Introduction

Depression is a frequent comorbidity in patients with heart failure (HF) [1]. Data from meta-analysis have shown that patients with HF have a mean depression prevalence of 21.5%, 2 to 3 times the rate of the general population [2]. Depression often exacerbates symptoms of patients with HF, doubling the risk of mortality and other cardiac events among patients with HF [3]. It is also a strong predictor of future cardiac events related to HF, similar to traditional cardiovascular risk factors [4]. Therefore, routine screening for and treatment of depression is an urgent need for patients experiencing HF. It is also suggested by the European Society of Cardiology (ESC) and American College of Cardiology/American Heart Association (ACC/AHA) HF guidelines [5, 6].

Review articles are now suggesting the therapeutic benefits of mind-body exercise on depression of varying severity [7–11]. Moreover, for patients with HF experiencing depression, interventions designed to improve both physical and psychological symptoms have been shown to reduce depression, increase physical function, and improve quality of life [12]. Thus, mind-body exercise may be the optimal therapy for depressive patients with HF.

TaiChi and Qigong are common mind-body exercises that originated in China. Each is an ancient Chinese healing art with a history of thousands of years [13, 14]. Qigong is an umbrella term covering a spectrum of mind-body exercises, such as Dao-Yin-Shu (physical and breathing exercise), Wu-Qin-Xi (five-animal play), Ba-Duan-Jin (eight-section health exercise), and Yi-Jin-Jing (changing tendons exercise) [15]. It is characterized by simple physical movements and is thought to be the grandfather to TaiChi, while TaiChi derives from Qigong and Martial Arts, involving more complex and choreographed movements [16]. Both TaiChi and Qigong employ slow and gentle physical movements, synchronized with breathing regulation and meditation to stretch the body, relax the muscles, coordinate breathing, and regulate attention and consciousness [17, 18]. Traditional Chinese medicine physicians believe that performing the meditative and synergistic dance-like movements of TaiChi and Qigong can promote Qi to flow harmoniously, thus promoting health and spirit [19].

Review articles and meta-analyses have demonstrated TaiChi's and Qigong's benefits on depression in the general population [10, 20], patients with chronic illnesses [9, 21–23], and patients with cardiovascular disease [24, 25]. Of note, TaiChi has served as a treatment for depression in many clinical trials, and evidence has shown that it is correlated with significant reductions in depression [18]. However, analysis for each illness has been limited to a handful of studies, precluding robust testing of whether the effects of TaiChi and Qigong differ by illness.

In recent years, many randomized controlled trials (RCTs) have reported the effects of TaiChi and Qigong among patients with HF, some of them reporting depression outcomes [26–33]. However, the results have been inconclusive, and convincing quantitative evidence to estimate treatment effects has been lacking. Therefore, this review evaluates the evidence from RCTs of TaiChi and Qigong on depression in patients with HF.

## 2. Methods

This systematic review was conducted and reported in accordance with the Preferred Reporting Items for Systematic Reviews and Meta-Analyses (PRISMA) statement [34] and the Cochrane Handbook for Interventional Reviews [35]. The study protocol has been published in PROSPERO (CRD42018081982). We followed the methods of Chen et al. (2020) [36].

### 2.1. Search Strategy

PubMed, Cochrane Central Register of Controlled Trials (CENTRAL), EMBASE, CINAHL, and China National Knowledge Infrastructure (CNKI) were searched from inceptions until October 23, 2019, without language restriction. Search terms were classified in three groups (Appendix 1: search strategy): condition (CHF, chronic heart failure and related terms), intervention (tradition Chinese exercise and related terms such as traditional exercise, mind-body exercise, Qigong, Taiji, Tai ji, Tai Chi, TaiChi, Liuzijue or Liu Zi Jue, or Baduanjin or Ba duan jin, or Wuqinxi or Wu Qin Xi, or Yijinjing, or Yi Jin Jing), and study type (RCT and related terms).

### 2.2. Study Selection

We applied the following selection criteria: (1) study design: RCTs reported in a full text; (2) participants: patients diagnosed with HF with or without restriction of left ventricular ejection fraction and in a stable phase of the disease; (3) interventions and control: RCTs comparing TaiChi and/or Qigong (TQPs) plus routine management (RM) with RM alone, or comparing TQPs plus RM with general exercise plus RM. RM included standard medical treatment, education, health guidance, or aerobic exercise. RCTs in which the intervention group was TQPs plus other traditional Chinese medicine therapies, such as acupuncture, Chinese herbal medicine preparation, were excluded; and (4) outcomes: depression or depressive symptoms measured by using patient-reported outcomes (PROs) or non-PRO assessment instruments.

After screening titles and abstracts from articles found in the searches, two reviewers (WJ, SL) retrieved potentially relevant full-text articles. These two reviewers independently assessed the eligibility of the full-text articles according to the selection criteria. Meanwhile, they resolved any discrepancies by agreement after rechecking the source articles or/and after discussion with a third reviewer (XC).

### 2.3. Data Extraction

One reviewer (WJ) extracted data with a standardized form from the included articles. A second reviewer (SL) checked the extracted data. Discrepancies were resolved by consensus after rechecking the source papers and further discussion with a third reviewer (XC).

The extracted data including study characteristics (e.g., title, author, publication year, and the use of randomization, allocation concealment, blinding and control, and country), participant characteristics (e.g., age, sex, sample size, New York Heart Association (NYHA) classification, and left ventricular ejection fraction (LVEF)), description of interventions (e.g., types of exercises, frequency, and durations) and controls, and instruments used to measure depression or depressive symptoms. Attempts were made to contact the original investigators regarding any missing data.

### 2.4. Risk of Bias Assessment

The trials' methodological quality was independently evaluated by two reviewers (WJ and SL) using the Cochrane risk of bias assessment tool. Discrepancies were resolved by agreement after rechecking the source papers and further discussion with a third reviewer (XC). Two reviewers assessed a quality rating for the following domains for each included trial as high risk, low risk, or uncertain risk of bias: (1) random sequence generation; (2) allocation concealment; (3) blinding of participants and personnel; (4) blinding of outcome assessors; (5) incomplete outcome data; and (6) selective reporting.

### 2.5. Data Analysis

The data from the included studies were analysed with RevMan 5.3 and STATA 12. Heterogeneity was assessed using a chi-square test (a *P* value <0.10 was considered indicative of statistical significance) and an *I*^2^ statistic (where *I*^2^ > 30%, 50%, or 75% indicated moderate, substantial, and considerable heterogeneity, respectively). Then, data from each trial were pooled with a random effects model in order to provide the included studies with more uniform weight. Given that all variables in the included studies consisted of continuous data and that various instruments were used, we used standardized mean difference (SMD) with 95% confidence intervals (95% CI) to analyse the outcomes. For studies with more than 2 control groups [33], such as TQPs plus RM vs. general exercise plus RM vs. RM alone, the means and standard deviations (SDs) of the two controls were combined using the methods described in the Cochrane Handbook (Section 6.5.2.10). A *P* value <0.05 was considered statistically significant.

Sensitivity analysis was first conducted by removing each study individually to estimate the results' consistency, as well as to explore the heterogeneity contributed from each individual study. Thereafter, subgroup analysis was undertaken to investigate the role of the various study characteristics on the observed effect, as well as to identify any potential sources of heterogeneity [37].

For trials with missing information on the means or SDs of outcomes, data were first sought from the original investigators. If it was not available from the author, then imputations were performed using the statistical approaches recommended in the Cochrane Handbook. One study used the median and interquartile ranges (IQR) instead of means and SDs [31]; the medians were taken as a substitute for the means and the SD was approximated as SD = IQR/1.35. In another study, SDs were imputed from a reported confidence interval [28] with missing SDs. Publication bias was not assessed due to the limited number of studies (<10) included in each analysis.

## 3. Results

**Figure 1:**
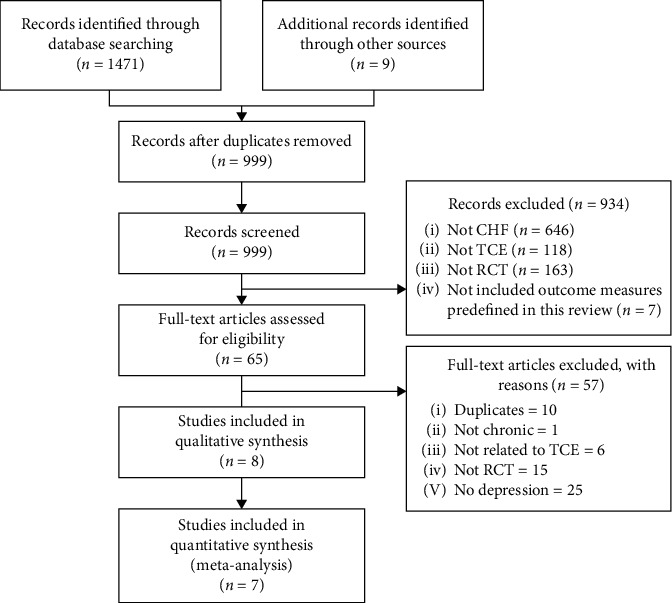
Search strategy and flow chart of the screened, excluded, and analysed articles. CHF: heart failure, TCE: traditional Chinese exercise, and RCT: randomized controlled trial.

1480 records (PubMed (*n* = 31), EMABSE (*n* = 64), Cochrane (*n* = 156), CINAHL (*n* = 29), CNKI (*n* = 1191)) totally were retrieved from the database searches. After excluding duplicates, 999 potentially relevant abstracts were screened, and 934 were excluded for failing to meet the inclusion criteria. The remaining 65 full texts were read, and finally, 8 RCTs [26–33] were deemed eligible f*or thi*s review.  Figure 1 presents reasons for exclusion.

### 3.1. Characteristics of the Included Studies

#### 3.1.1. Study Characteristics

Table 1 shows the main characteristics of selected studies. Eight RCTs (3 in Chinese [26–28] and 5 in English [29–33]) were published between 2007 and 2019. Regions of publication were the United States (*n* = 4) [30–33], Mainland China (*n* = 2) [26, 27], Taiwan (*n* = 1) [28], and the United Kingdom (*n* = 1) [29].

#### 3.1.2. Participants

Sample size per RCT ranged from 16 to 113, with a total of 514 patients who were elderly (mean age: 65 to 68 yrs.). The percentage of males ranged from 50% to 88%. Most patients were in NYHA classes II and III, and with LVEFs <40%, while two trials included patients with LVEFs ≥ 40% [32, 33].

#### 3.1.3. Intervention and Control

The majority of the trials used TaiChi (*n* = 6) [26, 27, 30–33], and the rest used Qigong (Chan-Chuang) [28] or TaiChi plus Qigong [29]. Exercise times lasted from 15 to 60 minutes per session. The length of exercise programs was either 12 weeks (*n* = 5) [26, 28, 30–32], 16 weeks (*n* = 2) [29, 33], or 24 weeks (*n* = 1) [27]. The exercises were center‐based in 5 trials [29–33], and home‐based in 3 trials [26–28]. The controls received the typical care, including medication and education advice in all trials and also received formal aerobic exercise training [32] or resistance band exercise [33].

#### 3.1.4. Outcome Measurement Instruments

The included studies used various depression severity scales (Table 2). This included three depression specific scales: the Hamilton Rating Scale for Depression (HAM‐D) [26, 27], the Beck Depression Inventory (BDI) [30–33], and the Hospital Anxiety and Depression Scale (HADS) [28]. The HAM‐D was assessed by clinicians while BDI and HADS were self-rated scales. Two general instruments were used and depression was reported as subscale scores: the Symptom Checklist 90-Revised (SCL-90R) [29] and the Profile of Mood States (PMOS) full [31] and brief versions [32]. Both instruments were self-rated scales.

#### 3.1.5. Depression Status

Based on the baseline mean scores and the reference cutoff points of the depression severity scales (Tables 1 and 2), participants were mildly depressed in four studies [28–30, 33] and moderately depressed in two studies [26, 27]. The other two studies using PMOS reported that 30% [31] and 37% [32] of the subjects had depression as a comorbidity, respectively. However, it was difficult to determine the average severity of the included participants as neither the classification nor the cutoff points of the PMOS scale were reported. Only one study applied clinically diagnosed depression as inclusion criteria for participants [26].

### 3.2. Methodological Quality of the Evidence

Table 1 presents the risk of bias assessments for individual studies (Appendix 3: risk of bias analysis). Five [26, 28, 31–33] out of eight RCTs described the methods of randomization. However, only two of the included trials [28, 33] reported allocation concealment details. Blinding of participants and personnel were judged as high risk of bias for most of the trials [26–30] due to the nature of the intervention. Blinding of outcome assessors was judged as high risk of bias for patient-reported scales. Two [26, 27] claimed that statisticians had been blinded. Most of the articles showed a low risk of incomplete outcome bias [26, 27, 29, 31–33]. Selective reporting bias was unclear in most RCTs [26–31, 33] because neither protocol nor trial registration information was available.

### 3.3. Outcomes

One study, conducted by Redwile et al. (2012) [30], could not be incorporated into the meta‐analysis because of the incomplete data presented. This study included 28 elderly patients (mean age 68 yrs.) undertaking 12-week center-based TaiChi training (*n* = 16) or typical care (*n* = 12). The author reported that patients in the TaiChi group had experienced reduced BDI total symptom scores from pre- to postintervention, compared to the controls. However, depression values were only provided at baseline, but not at the 12^th^ week. The remaining 7 trials reported various outcomes, including measurements of symptoms and depression status, which were included in the quantitative synthesis.

#### 3.3.1. Overall Effects

**Figure 2:**
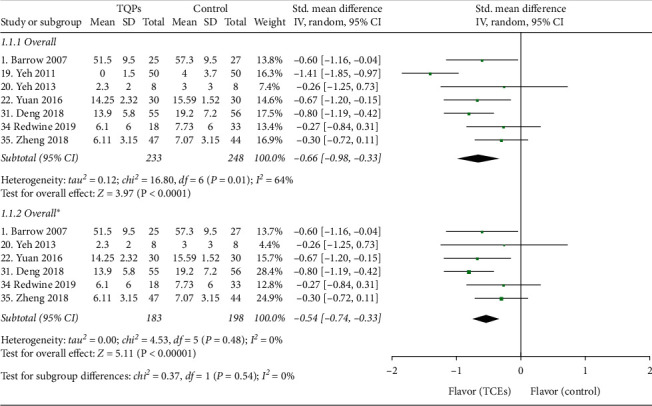
Meta-analysis results of overall pooled effects.

**Figure 3:**
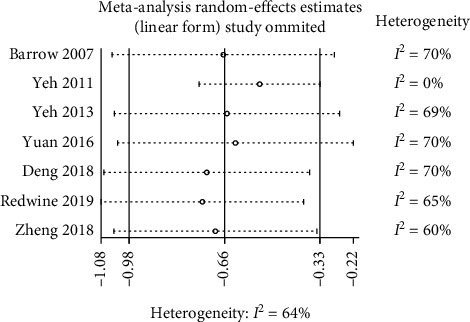
Influence of each individual study on the overall pooled effect estimate.

Pooling across the remaining seven RCTs (481 patients) provided evidence of a decrease in the depression symptoms with TQPs, but this analysis demonstrated substantial heterogeneity (SMD −0.66, 95% CI −0.98 to −0.33, *P* < 0.0001; *I*^2^ = 64%; Figure 2; Appendix 2: subgroup analysis (overall pooled effect)). Here a sensitive analysis was performed with one study removed at a time to explore potential sources of heterogeneity, as well as to assess whether the result could have been affected markedly by a single study. The result showed that Yeh et al.'s (2011) study [31] was removed, the statistical heterogeneity disappeared, and the pooled results continued to significantly favor the TQPs with smaller effects (SMD −0.54, 95% CI −0.74 to −0.33, *P* < 0.00001; *I*^2^ = 0%; Figure 2; Appendix 2: subgroup analysis (overall pooled effect)). However, omitting other studies altered neither effect estimates nor heterogeneity (Figure 3).

#### 3.3.2. Subgroup Analysis

We conducted subgroup analysis to investigate the role of the various study characteristics on the pooled effects, as well as to provide estimates of treatment effects for clinically relevant subgroups.

**Figure 4:**
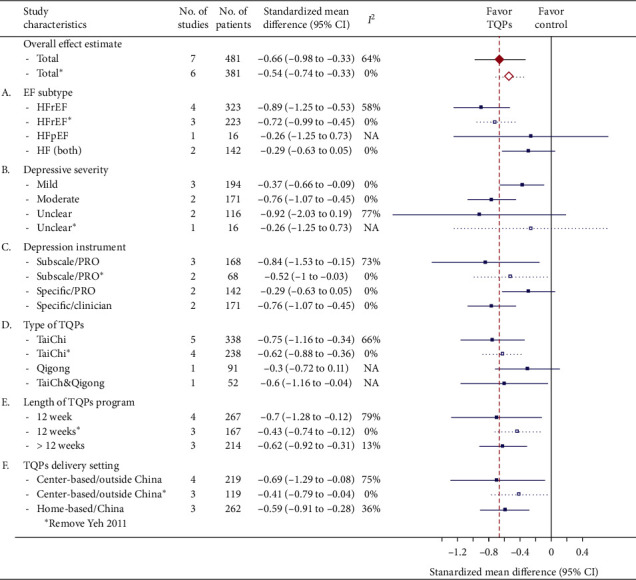
Influence of various study characteristics on the pooled effect and their contributions to heterogeneity. ^a^Details of meta-analysis results showing individual study data are presented in Appendix 2.

*(1) Participants' EF*. The benefits of TQPs on depressive symptoms according to ejection fraction (EF) subtype were inconsistent (Figure 4(a); Appendix 2: subgroup analysis (EF subtypes)). The pooled results favored TQPs with increased effect size and remained significant for the heart failure with reduced ejection fraction (HFrEF) RCTs. However, when pooling the two trials including patients with both HFrEF and heart failure with preserved ejection fraction (HFpEF), an insignificant beneficial effect towards the TQPs was found.

*(2) Depressive Severity*. In the subgroup analysis for participants' depressive severity, the moderately depressed patients benefited more than the mildly depressed patients (SMD −0.76 versus −0.37; Figure 4(b); Appendix 2: subgroup analysis (depressive severity)). However, when pooling the two trials with unknown depression severity, the beneficial effects became insignificant.

*(3) Depression Instruments*. The pooled results were inconsistent across subgroups according to the depression instruments (Figure 4C; Appendix 2: subgroup analysis (depression instrument)). When pooling two trials in which the depression severity was evaluated by the clinicians, the beneficial effects from TQPs exceeded the overall effects (Figure 4(c): clinician; Appendix 2: subgroup analysis (depression instrument)). However, when pooling two trials in which patients self-rated their depressive severity using depression specific instruments (BDI and HADS), the beneficial effects from TQPs became smaller and insignificant (Figure 4(c): PRO/specific; Appendix 2: subgroup analysis (depression instrument)). Of note, when pooling the other three trials in which depressive severity was also self-rated by patients, but using nondepression specific instruments (PMOS and SCL-90R), the pooled results became significant again with a larger effect (Figure 4(c): PRO/nonspecific; Appendix 2: subgroup analysis (depression instrument)).

*(4) Characteristics of TQP Programs*. Generally, the pooled effects were not influenced by the characteristics of the TQP programs. There were similar pooled effect sizes in the *TaiChi* subgroup (Figure 4(d); Appendix 2: subgroup analysis (TQPs)). In addition, TQP program length (<12 weeks vs. >12 weeks) might not have influenced the pooled effect sizes (Figure 4(e); Appendix 2: subgroup analysis (length of TQP programs)). Furthermore, the effect sizes were also similar whether the TQPs were delivered at centers or were home-based (Figure 4(f); Appendix 2: subgroup analysis (TQP delivery settings)). As the sensitivity analysis showed that the study by Yeh et al. (2011) [31] was the main contributor to the heterogeneity (Figure 3), we removed this study and then repeated the subgroup analysis. This change solved the statistical heterogeneity and resulted in a mitigated pooled effect, but did not alter the (in)significance of the pooled results in the associated subgroups (dashed line in Figure 4).

### 3.4. TQP Safety and Overall Evidence

Patient dropout in the TQP groups was low, with most withdrawals being due to hospitalization or CHF exacerbation. We found no adverse events related to TQPs in the included studies.

## 4. Discussion

To our knowledge, this is the first systematic review to synthesize RCTs written in both Chinese and English languages that have explored the potential effects of TaiChi and Qigong on depression among patients with HF. Eight RCTs were included, and seven RCTs involving 481 patients with HF were evaluated in the meta-analysis. The results showed that practicing TaiChi and Qigong were associated with a significant reduction in depressive symptoms (SMD −0.66, 95% CI −0.98 to −0.33, *P* < 0.0001; *I*^2^ = 64%), and its antidepressive effect was not influenced by intervention duration or exercise setting, but rather by EF subtype, depressive severity, and depression instruments. Significant effects were found for HFrEF (SMD: −0.89; 95% CI −1.25 to −0.53; *I*^2^ = 54%), moderate depression (SMD: −0.76; 95% CI −1.07 to −0.45; *I*^2^ = 0%), mild depression (SMD: −0.37; 95% CI −0.66 to −0.09; *I*^2^ = 0%), specific clinician-rated scales (HAM-D) evaluated depression severity (SMD: −0.76; 95% CI −1.07 to −0.45; *I*^2^ = 0%), and nonspecific self-rated scales (PMOS and SCL-90R) evaluated depression severity (SMD: −0.84; 95% CI −1.53 to −0.15; *I*^2^ = 73%); however, not for HFrEF and HFpEF, specific self-rated scales (BDI and HADS) measured depressive severity. In addition, the beneficial effects of TQPs were preserved when we removed the study with the largest effect (SMD −0.54, 95% CI −0.74 to −0.33, *P* < 0.00001; *I*^2^ = 0%).

Our review is consistent with other systematic reviews and meta-analysis supporting the fact that TaiChi and Qigong reduce depressive symptoms [9, 10, 21]. One review of 17 randomized controlled or nonrandomized trials found that TaiChi reduced depression by heterogeneous standardized effects of −0.66 (95% CI −0.29 to −1.03) among various populations [10]. Gu et al. [38] reviewed evidence on TaiChi in relation to various clinical outcomes including depressive symptoms among patients with HF, founding that TaiChi resulted in significant depression-reduction effects, compared to the control. However, this result was based on only two RCTs involving 112 participants from the USA, most of whom were white. Unlike these previous studies, our review provides a synthesis of the evidence on all types of TaiChi and Qigong with a focus on patients with HF. In addition, previous studies have suggested that emotional responses to TaiChi or Qigong may vary across cultures [39]. Through extensive literature search, our review included the most updated evidence from Mainland China, Taiwan, the USA, and the UK, covering a range of ethnic groups.

The mechanism whereby traditional Chinese Qigong attenuates symptoms of depression is probably multifactorial. An underlying philosophy of the practice is that any form of traditional Chinese Qigong has an effect on the cultivation of balance and the harmony of vital energy (Qi), which functions as a holistic, coherent, and mutually interactive system [18]. In terms of biological mechanisms, it has been reported that the activity and connectivity of key brain regions related to depression, the autonomic nervous system, and neuroinflammatory sensitization can be modulated by TaiChi and Qigong [18]. Another proposed mechanism suggests that the antidepressant effects of TaiChi and Qigong are associated with improvement in other clinical outcomes such as functional capacity and quality of life [18]. Measures of psychological variables and a multitude of other outcome measures are empirically interrelated, and treatment of each outcome can reciprocally and exponentially improve the other. Additionally, the mutual encouragement and friendly companionship from peers due to the collective activities of TQPs benefit the cognitive control network, adding to the effects related to the TaiChi or Qigong intervention [23].

### 4.1. Implications for Future Research

Although promising, several knowledge gaps identified in the present review need rational consideration and emphasis in future research. Data from the present review suggests favorable effects of TQPs on depressive symptoms among patients with HF, but this effect is restricted to depressed patients with HFrEF; it is not presented among depressed patients with HFpEF. The negative effects of TQPs on depressive symptoms in HFpEF remain to be determined because there was only one study [32] reviewed in our article regarding TQPs' effects on HFpEF, and it had a small sample size (*n* = 16). These findings accord with a previous meta-analysis assessing exercise training for patients with HF and comorbid depression in which only three trials were available regarding the antidepressive effect of exercise training on HFpEF [40].

There is no denying that insufficient samples partially explain the limited benefits experienced by patients with HFpEF and depression. Therefore, future large-scale multicenter RCTs with a sufficient number of participants are needed to verify TQPs' positive effects among this population. Likewise, the antidepressive effect of TQPs for patients with HF and comorbid major depression is less established. In our review, the available data describe the improvement due to TQPs in minor and moderate depression, and even depression without clear severity in patients with HF, except for major depression. Prior work [12] has emphasized that patients with HF combined with various depression severity have significant differences in the patterns of studies' findings of primary outcomes, such as depression, physical function, and quality of life. This suggests that the findings should be dichotomized according to depression severity to clarify the intervention effects. For this reason, it would be unreasonable to speculate TQPs' benefit on major depression based on its antidepressive effect on minor or moderate depression. Future studies need to enroll patients with HF whose major depression can be diagnosed definitively to evaluate TQPs' antidepressive effect.

In terms of TQPs' antidepressive effect, the present review indicates that it varies depending on the depression instruments used. In this light, it would be informative to consider whether and how to choose suitable depression instruments based on patient population, depression severity, and study setting when evaluating the intervention effects. Thus far, at least two instruments beyond those mentioned in our review have been widely applied to studies to detect and quantify depression in HF, i.e., the 9-item Patient Health Questionnaire (PHQ-9) [41] and the Montgomery and Asberg Depression Rating Scale (MADRS) [42]. However, many studies, including influential trials such as the HF-ACTION [43], the MOOD-HF [42], and the SADAHRT-CHF [44] do not state the usage reasons for a specific instrument. Thus, their results are less convincing. The 21-item Beck Depression Inventory (BDI-II) and the PHQ-9 are the more mature instruments and are recommended in HF combined with depression [43, 45, 46]. In view of a lack of specific guidelines for using depression instruments in HF, hopefully future clinical trials using BDI-II and PHQ-9 will accurately measure depressive symptoms and disorders in patients with HF.

In addition, data indicated that Qigong, or TaiChi combined with Qigong, is less promising than TaiChi alone for depressed patients with HF. This leaves TaiChi as the state-of-the-art mind-body exercise for heart failure and depression in Chinese medicine. Yet definitive answers are missing, and the most effective type of intervention remains to be determined. According to our systematic review, to date there has only been one trial [28] with a small sample reporting changes in depressive symptoms with Qigong practice. This is insufficient to conclude that patients with HF suffering from depression do not respond well to Qigong. Moreover, the reduction in Qigong's comparative disadvantage to TaiChi was achieved indirectly by comparing to control groups. The results of this analysis are similar to another meta-analysis on traditional Chinese exercises (TCEs) for depression accompanying cardiovascular disease [24], which failed to investigate the benefits of specific TCEs to identify the ideal intervention. The situation for TaiChi and Qigong [29] is similar. In this light, there is an unmet need in HF for sufficiently powered trials of TQPs' effect on depression remission. Moreover, a larger-scale RCT on Qigong, an RCT to compare TCEs, and a network meta-analysis comparing TCEs could fill this research gap, thus determining the optimal TCEs to treat depression in HF.

Furthermore, data existing fails to demonstrate differences between the TQPs' effects compared with general exercise on depression in patients with HF. A recent systematic review and network meta-analysis on clinical depression in older adults [47] pointed out that mind-body exercise showed the largest improvement on depressive symptoms, followed by aerobic exercise and resistance exercise, despite a lack of statistically significant differences. Nevertheless, the results only provide limited insight into TQPs for depression in HF. In line with the present review, at this stage, we can only speculate on the relative benefits of TQPs based on two studies. These studies compared the antidepressive effect between TaiChi and aerobic exercise and between Qigong and resistance band training. Therefore, well-designed RCTs are needed to verify the relative benefits of TQPs for depression in HF to general exercise, including aerobic exercise and resistance exercise. This could promote a more solid foundation for treatment recommendations.

However, data from our review have demonstrated how TQPs mitigate depressive symptoms, but the benefits in clinical outcomes such as hospitalization and mortality remain uncertain. Logic would allow one to assume that treating depression may decrease poor outcomes in patients with HF, given that depression can increase the risk of poor outcomes for patients experiencing HF. For example, the HF-ACTION trials [43] showed that exercise benefits patients with HF. Its ancillary study documented that reduced depressive symptoms were associated with improved clinical outcomes. This provided valuable insight into the screening and exercise treatment of depression in patients with HF. Thus, future studies should be designed to determine whether reduced depressive symptoms are associated with improved clinical outcomes, in addition to assessing the effects of TQPs on depressive symptoms.

### 4.2. Limitations

Several limitations in the present analysis should be noted. First, our analysis of the depression data symptoms was restricted to an SMD, as numerous depression scales were used in the included trials. Therefore, the results should be interpreted with this in mind. Another limitation is the presence of statistical heterogeneity between the trials in this meta-analysis. In order to provide the studies with more uniform weight, we used a random effects model. A sensitivity analysis and subgroup analysis were also performed to investigate the heterogeneity. In our meta-analysis, the beneficial effects [31] were preserved without heterogeneity when the study with the largest effect was removed. Finally, our conclusions were constrained by the quality of the trials reviewed. The main shortcomings were the lack of blinding procedures. However, in exercise interventions, double-blinding is not feasible without deception. In addition, most of the instruments used were self-rated scales where blinding the outcome assessors, i.e., the patients themselves, would have been impossible. These two features could have led to favorable responses among the participants in the intervention group. Therefore, blinding statisticians is essential in this type of trial, but only one included trial provided this information. The absence of randomization concealment and of the intention-to-treat (ITT) analyses is another concern when interpreting the results. Publication bias, although not evaluated, might have been present in the included studies since positive trials are more likely to be published than negative trials. Hence, the effect sizes might have been overestimated. Finally, many studies have neglected critical information in terms of allocation concealment, outcome assessor blinding, adequate follow-up, and ITT analysis. Future RCTs are warranted to improve the methodological quality by adhering to the Consolidated Standards of Reporting Trials statement (CONSORT) [48] or its extensions [49].

## 5. Conclusion

The evidence presented in this review should encourage physicians to recommend TQPs as clinically effective ways to reduce depressive symptoms in patients with HF. Additional RCTs with rigorous research design, which focus on the above research gaps, are warranted to establish the therapeutic effects of TQPs for depression in HF.

## Figures and Tables

**Table 1:** Characteristics of included studies.

Source (country)	Populations				Types of TQPs (time/frequency; duration)	Control	RM^a^	Depression severity			Risk of bias^b^
	NYHA subtype	Sample size (drop out) (I/C), #	Male (I/C), %	Age, yrs. (I/C) mean ± SD				Instrument	Baseline mean (I/C)	Changes mean (I/C)	
Barrow et al. (2007) [29] (UK)	II∼III HFrEF	65 (32/33) (13 (7/6))	81%/82%	68.4 ± NA^c^/67.9 ± NA^c^	III. TaiChi & Qigong (55 mins/twice per week; 16 wks)	—	TDs	SCL-90R^d^, DEP subscale	Mild 58.3/60.2	−6.8/−2.9 (neutral)^e^	Unclear; unclear; high; high; low; unclear
Redwine et al. (2012) [30] (US)	II HFpEF & HFrEF	24 (12/12) (4 (4/0))	83%/92%	72.6 ± 6.2/63.9 ± 12.0	I. TaiChi (60 min/twice per week; 12 wks)	—	TDs	BDI^d^, DEP specific	Mild 8.0/9.2	NA/NA^c^ (positive)^f^	Unclear; unclear; high; high; unclear; unclear
Yeh et al. (2011) [31] (US)	I∼III HFrEF	100 (50/50) (4 (1/3))	56%/72%	68.1 ± 11.9/66.6 ± 12.1	I. TaiChi (60 min/twice per week; 12 wks)	Education	TDs; dietary, exercise advice	PMOS-full^d^, DEP subscale	Unclear^g^ 2.0/3.0	−2.0/1.0 (neutral)^e^	Low; unclear; unclear; unclear; low; high
Yeh et al. (2013) [32] (US)	I∼III HFpEF	16 (8/8) (0)	50%/50%	68.0 ± 11.0/63.0 ± 11.0	I. TaiChi (60 min/twice per week; 12 wks)	Aerobic exercise^h^	TDs; dietary, exercise advice	PMOS-brief^d^, DEP subscale	Unclear^i^ 4.0/1.3	−1.7/+1.7 (*p*=0.05)^e^	Low; unclear; low; low; low; unclear
Yuan et al. (2016) [26] (China)	II∼III HFrEF	60 (30/30) (0)	57%/53%	66.3 ± 5.6/67.5 ± 3.8	I. TaiChi (20–40 min/5 times per week; 12 wks)	—	TDs; education; antidepressants	HAM-D^d^, DEP specific	Moderate^j^ 19.9/19.5	−5.6/−3.9 (positive)^e^	Low; unclear; high; low; low; unclear
Deng et al. (2018) [27] (China)	I∼III HFrEF	113 (57/56) (2 (2/0))	54%/52%	64.7 ± 4.2/67.2 ± 4.9	I. TaiChi (40∼60 min/≥5 times per week; 24 wks)	—	TDs; daily life advice	HAM-D^d^, DEP specific	Moderate 22.6/21.3	−8.7/−2.1 (positive)^e^	Unclear; unclear; high; unclear; low; unclear
Redwine et al. (2019) [33] (US)	NA^d^ HFrEF & HFpEF	45 (25/23/22) (7 (4/0/3))	92%/86%/87%	63.0 ± 9.0/67.0 ± 7.0/65.0 ± 9.0	I. TaiChi (60 min/twice per week; 16 wks)	−/resistance band^k^	TDs; usual care	BDI^d^, DEP specific	Mild 9.6/8.0/11.9	−3.5/−1/−3.3 (positive)^f^	Low; low; unclear; unclear; low unclear
Cheng et al. (2018) [28] (Taiwan)	II NA^c^	91 (41/44) (9 (3/6))	72%/70%	62.2 ± 15.1/66.6 ± 12.7	II. Qigong (Chan-Chuang) (≥15 min/2∼3 times per day; 12 wks)	—	TDs	HADS^d^, DEP specific	Mild 7.2/7.3	−1.1/−0.2 (positive)^f^	Low; low; high; high; unclear; unclear

NYHY: New York Heart Association; I: intervention group; C: control group; SD: standard deviation; TQPs: TaiChi and/or Qigong practices; RM: routine management; HFrEF: heart failure with reduced ejection fraction; HFpEF: heart failure with perceived ejection fraction; NA: not available; wks: weeks; TDs: therapeutic drugs (prescribed according to heart failure management guideline); DEP: depression; SCL-90-R: Symptom Checklist-90-Revised; BDI: Beck Depression Inventory; PMOS: Profile of Mood States; HAM-D: Hamilton Rating Scale for Depression; HADS: Hospital Anxiety and Depression Scale. ^a^Routine management provided as a consistent cointervention to both groups. ^b^Risk of bias tool domains: (1) random sequence generation; (2) allocation concealment; (3) blinding of patients and personnel; (4) blinding of outcome assessors for primary outcomes; (5) incomplete outcome data; (6) selective reporting, respectively. ^c^LVEF was not measured. ^d^Lower sum scores denote improvement. ^e^Between-group comparisons. ^f^Group-by-time interaction. ^g^The classification or the cutoff points of the scale (PMOS) were not found, but 30% of the subjects had depression as a comorbidity. ^h^Aerobic exercise: 60 min/twice per week. ^i^The classification or the cutoff points of the scale (PMOS) were not found, but 37% of the subjects had depression as a comorbidity. ^j^Clinically diagnosed depression according to the CCMD-3 classification scheme and diagnostic criteria of Chinese psychosis. ^k^Resistance band training: 60 min/twice per week.

**Table 2:** Summary of the depression severity scales used in the included studies.

Instruments (no. of study)	Objective	Rater; number of item; rating scale	Categorization/cutoff
SCL-90R, DEP subscale (*n* = 1) [29]	To reflect the psychological symptom patterns in 9 domains: somatization/obsessive-compulsive/sensitivity/depression/anxiety/hostility/phobic anxiety/paranoid ideation/psychoticism	PRO; 90 items (DEP: *n* = 13); 5-point scale (0∼4)^a^	A T-score^b^ ranging from 40 to 60 represents the normal range^c^
BDI, DEP specific (*n* = 2) [30, 33]	To measure the severity of depression in adults and adolescents, two subscales include a cognitive-affective subscale and a somatic-performance subscale	PRO; 21 items; 4-point scale (0∼3)^a^	0–13: minimal; 14–19: mild depression; 20–28: moderate; 29–63: severe^d^
			In nonclinical populations, scores above 20 indicate depression

PMOS-full, DEP subscale (*n* = 1) [31]	To assess emotional states in 6 domains: depression/anxiety/fatigue/vigor/irritability/confusion	PRO; 65 items (DEP: *n* = 15); 5-point scale (0∼4)^a^	Not found
PMOS-brief DEP subscale (*n* = 1) [32]	Same as the full version	PRO; 30 items (DEP: *n* = 5); 5-point scale (0∼4)^a^	Not found
HAM-D DEP specific (*n* = 2) [26, 27]	The “gold standard” for assessing severity of depressive severity	Clinician; 17 items; 5-point scale (0–4)^a^ (*n* = 8); 3-point scale (0–2)^a^ (*n* = 9)	0–7: normal; 8–16: mild; 17–23: moderate; 24–50: severe^e^
HADS DEP specific^f^ (*n* = 1) [28]	To assess anxiety and depression symptoms in medical patients	PRO; 14 items (DEP: *n* = 7); 4-point scale (0∼3)^a^	0–7: normal; 8–10: mild; 11–14: moderate; 15–21: severe^g^
			A cutoff of 8: clinically significant depression

PRO: patient-reported outcome; DEP: depression; SCL-90R: Symptom Checklist-90-Revised; BDI: Beck Depression Inventory; PMOS: Profile of Mood States; HAM-D: Hamilton Rating Scale for Depression; HADS: Hospital Anxiety and Depression Scale. ^a^Higher scores indicate depressed. ^b^The SCL-90-R scores are converted to standard *T*-scores (ranging from 30 to 80) by referring to the appropriate population-based norm tables provided by the test manual and a *T*-score of 50 represents the mean of the respective normal population. ^c^Holi, M. (2003). Assessment of psychiatric symptoms using the SCL-90. ^d^Jackson-Koku, G. (2016). Beck depression inventory. Occupational Medicine, 66 (2), 174-175. ^e^Zimmerman, M., Martinez, J. H., Young, D., Chelminski, I., & Dalrymple, K. (2013). Severity classification on the Hamilton Depression Rating Scale. Journal of Affective Disorders, 150 (2), 384–388. ^f^Although the anxiety and depression questions are interspersed within the questionnaire, it is vital that these are scored separately. ^g^Stern, A. F. (2014). The Hospital Anxiety and Depression Scale. Occupational Medicine, 64 (5), 393–394.

## Data Availability

The extracted data used to support the findings of this study are available from the corresponding author upon request.

## Abbreviations

BDI: Beck Depression Inventory
CI: Confidence intervals
EF: Ejection fraction
HADS: Hospital Anxiety and Depression Scale
HAM-D: Hamilton Rating Scale for Depression
HF: Heart failure
HFpEF: Heart failure with preserved ejection fraction
HFrEF: Heart failure with reduced ejection fraction
IQR: Interquartile ranges
ITT: Intention-to-treat
LVEF: Left ventricular ejection fraction
MADRS: Montgomery and Asberg Depression Rating Scale
NYHA: New York Heart Association
PHQ-9: 9-item Patient Health Questionnaire
PMOS: Profile of Mood States
PROs: Patient-reported outcomes
RCTs: Randomized controlled trials
RM: Routine management
SCL-90R: Symptom Checklist 90-Revised
SDs: Standard deviations
SMD: Standardized mean difference
TCEs: Traditional Chinese exercises
TQPs: TaiChi and/or Qigong practices.

## Supplementary Materials

Supplementary Materials Appendix 1: search strategy. Appendix 2: subgroup analysis. Appendix 3: risk of bias analysis. Appendix 4: PRISMA 2009 checklist.
