# Supplementary material for: TaiChi and Qigong for Depressive Symptoms in Patients with Chronic Heart Failure: A Systematic Review with Meta-Analysis
Source: Evid Based Complement Alternat Med. 2021 Jun 24;2021:5585239. doi: 10.1155/2021/5585239 (PMC8302391; doi:10.1155/2021/5585239)

Appendix

Appendix 1. Search strategy

PubMed search strategy

1. "Heart Failure"[Mesh] OR

2. "heart failure"[Title/Abstract] OR "cardiac failure"[Title/Abstract] OR "Myocardia failure"[Title/Abstract] OR "Ventricular failure"[Title/Abstract] OR "Heart decompensation"[Title/Abstract]

3. or1/2

4. "traditional exercise*"[Text Word] OR "chinese traditional exercise*" [Text Word] OR "traditional chinese exercise*"[Text Word] OR "chinese exercise*"[Text Word]

5. "taiji"[Title/Abstract] OR "tai ji"[Title/Abstract] OR "tai chi"[Title/Abstract] OR "qigong"[Title/Abstract] OR "qi gong"[Title/Abstract] OR "liuzijue"[Title/Abstract] OR "liu zi jue" [Title/Abstract] OR "wuqinxi"[Title/Abstract] OR "wu qin xi"[Title/Abstract] OR "yijinjing"[Title/Abstract] OR "yi jin jing"[Title/Abstract] OR "baduanjin"[Title/Abstract] OR "ba duan jin"[Title/Abstract] OR "eight section brocades"[Title/Abstract] OR "eight section brocades"[Title/Abstract]

6. or/4-5

7. "Randomized Controlled Trial" [Publication Type]

8. "Controlled Clinical Trial" [Publication Type]

9. randomized[Text Word]

10. placebo[Text Word]

11. randomly[Text Word]

12. trial[Text Word]

13. groups[Text Word]

14. or/7-13

15. ("Animals"[Mesh]) NOT (("Animals"[Mesh]) AND "Humans"[Mesh])

16. 14 not 15

17. 3 AND 6 AND 16

EMBASE search strategy

1. 'heart failure'/exp

2. "heart failure":ab,ti OR "cardiac failure":ab,ti OR "Myocardia failure":ab,ti OR "Ventricular failure":ab,ti OR "Heart decompensation":ab,ti

3. or1/2

4. "traditional exercise*" OR "chinese traditional exercise*" OR "traditional chinese exercise*" OR "chinese exercise*"

5. "taiji":ab,ti OR "tai ji":ab,ti OR "tai chi":ab,ti OR "qigong":ab,ti OR "qi gong":ab,ti OR "liuzijue":ab,ti OR "liu zi jue":ab,ti OR "wuqinxi":ab,ti OR "wu qin xi":ab,ti OR "yijinjing":ab,ti OR "yi jin jing":ab,ti OR "baduanjin":ab,ti OR "ba duan jin":ab,ti OR "eight section brocades":ab,ti OR "eight section brocades":ab,ti

6. or/4-5

7. 'randomized controlled trial (topic)'/exp

8. 'controlled clinical trial'/exp

9. randomized.ab.

10. placebo.ab.

11. randomly.ab.

12. trial.ab.

13. groups.ab.

14. or/1-7

15. 'animal'/exp not ('human'/exp and 'animal'/exp)

16. 14 not 15

17. 3 AND 6 AND 16

Cochrane Library search strategy

1. MeSH descriptor: [Heart Failure] explode all trees

2. "heart failure":ti,ab,kw OR "cardiac failure":ti,ab,kw OR "Myocardia failure":ti,ab,kw OR "Ventricular failure":ti,ab,kw OR "Heart decompensation":ti,ab,kw

3. or1/2

4. traditional exercise* OR chinese traditional exercise* OR traditional chinese exercise* OR chinese exercise OR

5. ("taiji"):ti,ab,kw OR ("tai ji"):ti,ab,kw OR ("tai chi"):ti,ab,kw OR ("qigong"):ti,ab,kw OR ("qi gong"):ti,ab,kw OR ("liuzijue"):ti,ab,kw OR ("liu zi jue"):ti,ab,kw OR ("wuqinxi"):ti,ab,kw OR ("wu qin xi"):ti,ab,kw OR ("yijinjing"):ti,ab,kw OR ("yi jin jing"):ti,ab,kw OR ("baduanjin"):ti,ab,kw OR ("ba duan jin"):ti,ab,kw OR ("eight section brocades"):ti,ab,kw OR ("eight section brocades"):ti,ab,kw

6. or/4-5

7. Randomized Controlled Trial:pt

8. Controlled Clinical Trial:pt

9. randomized:ti,ab,kw

10. placebo:ti,ab,kw

11. randomly:ti,ab,kw

12. trial:ti,ab,kw

13. groups:ti,ab,kw

14. or/7-13

15. ("Animals"[Mesh]) NOT (("Animals"[Mesh]) AND "Humans"[Mesh])

16. 14 not 15

17. 3 AND 6 AND 16

CINAHL search strategy

1. MH Heart Failure

2. TI("heart failure" OR "cardiac failure" OR "Myocardia failure" OR "Ventricular failure" OR "Heart decompensation")

3. AB("heart failure" OR "cardiac failure" OR "Myocardia failure" OR "Ventricular failure" OR "Heart decompensation")

4.or/1-3

5. TX ("traditional exercise*" OR "chinese traditional exercise*" OR "traditional chinese exercise*" OR "chinese exercise*")

6. TI ("taiji" OR "tai ji" OR "tai chi" OR "qigong" OR "qi gong" OR "liuzijue" OR "liu zi jue" OR "wuqinxi" OR "wu qin xi" OR "yijinjing" OR "yi jin jing" OR "baduanjin" OR "ba duan jin" OR "eight section brocades" OR "eight section brocades")

7. AB ("taiji" OR "tai ji" OR "tai chi" OR "qigong" OR "qi gong" OR "liuzijue" OR "liu zi jue" OR "wuqinxi" OR "wu qin xi" OR "yijinjing" OR "yi jin jing" OR "baduanjin" OR "ba duan jin" OR "eight section brocades" OR "eight section brocades")

8. or/5-7

9. MH("Random Assignment" OR "Placebos" OR "Placebo Effect" OR "Single-Blind Studies" OR "Double-Blind Studies" OR "Triple-Blind Studies" OR "Randomized Controlled Trials" OR "comparative studies" OR "Evaluation Research" OR "Prospective Studies" OR "crossover Design" OR "Prospective Studies" OR "Clinical Trials" OR "Clinical Trial Registry")

10. TX(random$ OR allocation OR "random allocation" OR placebo$ OR single blind OR double blind OR "randomi?ed controlled trial*" OR "controlled clinical trial*" OR "comparative study" OR "evaluation stud*" OR "follow-up stud*" OR "prospective stud*" OR "cross-over stud*" OR control$ OR prospectiv$ OR volunteer$ OR "RCT" OR "clinical trial*")

11. PT(randomized controlled trial OR "clinical trial*")

12. or/9-11

13. 4 AND 8 AND 12

CNKI search strategy in English CNKI search strategy

1. FT = randomize or FT = randomized allocation or FT = randomized controlled or FT = controlled or FT = blind or FT = single blind or FT = double blind or FT = randomized controlled trial or FT = randomized controlled study or FT = clinical trial or FT = clinical observation or FT = clinical study

2. SU = taijiquan or SU = qigong or SU = six-character formula or SU = yijinjing or SU = wuqinxi or SU = baduanjin or SU = traditional training or SU = traditional Chinese sports training or SU = Chinese training (exact match)

3. SU = cardiovascular disease or SU = coronary heart disease or SU = hypertension or SU = hyperlipidemia or SU = angina pectoris or SU = myocardial infarction or SU = arrhythmia or SU = heart failure or SU = heart failure or SU = myocardial ischemia or SU = atherosclerosis or SU = coronary artery disease or SU = hypertension

4. AND/1-

Appendix 2. Subgroup analysis

Overall pooled effect

Study or Subgroup	TQPs Mean	SD	Total	Control Mean	SD	Total	Weight	Std. Mean Difference IV, Random, 95% CI
**1.1.1 Overall**								
1. Barrow 2007	51.5	9.5	25	57.3	9.5	27	13.8%	−0.60 [−1.16, −0.04]
19. Yeh 2011	0	1.5	50	4	3.7	50	16.3%	−1.41 [−1.85, −0.97]
20. Yeh 2013	2.3	2	8	3	3	8	7.4%	−0.26 [−1.25, 0.73]
22. Yuan 2016	14.25	2.32	30	15.59	1.52	30	14.6%	−0.67 [−1.20, −0.15]
31. Deng 2018	13.9	5.8	55	19.2	7.2	56	17.5%	−0.80 [−1.19, −0.42]
34 Redwine 2019	6.1	6	18	7.73	6	33	13.4%	−0.27 [−0.84, 0.31]
35. Zheng 2018	6.11	3.15	47	7.07	3.15	44	16.9%	−0.30 [−0.72, 0.11]
**Subtotal (95% CI)**			**233**			**248**	**100.0%**	**−0.66 [−0.98, −0.33]**
Heterogeneity: $Tau^2 = 0.12$; $Chi^2 = 16.80$, df = 6 (P = 0.01); $I^2 = 64\%$								
Test for overall effect: Z = 3.97 (P < 0.0001)								
**1.1.2 Overall***								
1. Barrow 2007	51.5	9.5	25	57.3	9.5	27	13.7%	−0.60 [−1.16, −0.04]
20. Yeh 2013	2.3	2	8	3	3	8	4.4%	−0.26 [−1.25, 0.73]
22. Yuan 2016	14.25	2.32	30	15.59	1.52	30	15.7%	−0.67 [−1.20, −0.15]
31. Deng 2018	13.9	5.8	55	19.2	7.2	56	28.4%	−0.80 [−1.19, −0.42]
34 Redwine 2019	6.1	6	18	7.73	6	33	12.8%	−0.27 [−0.84, 0.31]
35. Zheng 2018	6.11	3.15	47	7.07	3.15	44	24.9%	−0.30 [−0.72, 0.11]
**Subtotal (95% CI)**			**183**			**198**	**100.0%**	**−0.54 [−0.74, −0.33]**
Heterogeneity: $Tau^2 = 0.00$; $Chi^2 = 4.53$, df = 5 (P = 0.48); $I^2 = 0\%$								
Test for overall effect: Z = 5.11 (P < 0.00001)								

Test for subgroup differences: $Chi^2 = 0.37$, df = 1 (P = 0.54), $I^2 = 0\%$

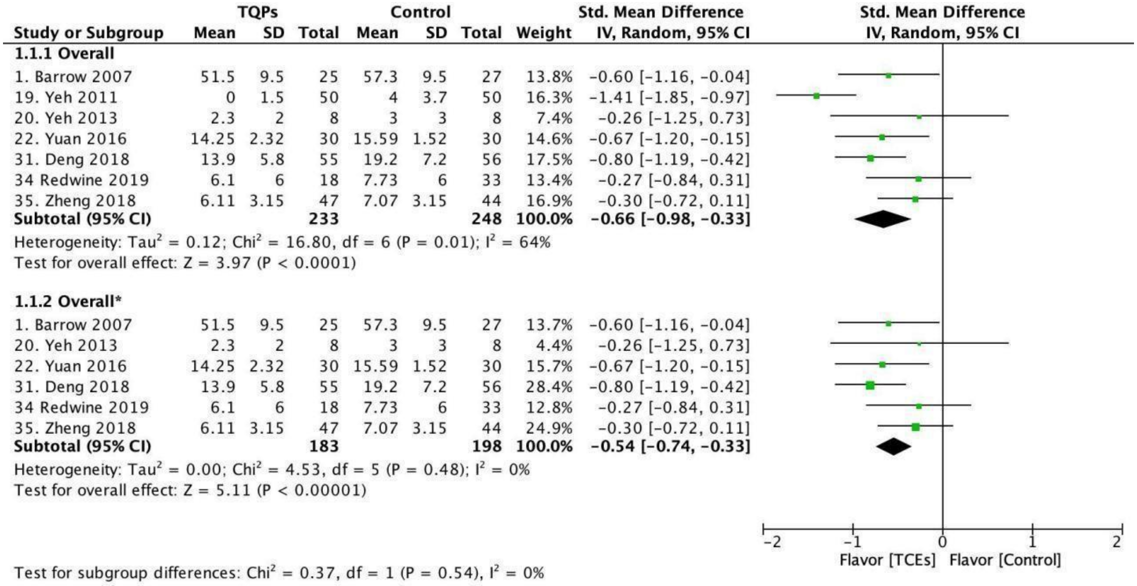

Subgroup analysis (EF Subtypes)

Study or Subgroup	TCEs Mean	SD	Total	Control Mean	SD	Total	Weight	Std. Mean Difference IV, Random, 95% CI
**1.2.1 HFrEF**								
1. Barrow 2007	51.5	9.5	25	57.3	9.5	27	21.5%	−0.60 [−1.16, −0.04]
19. Yeh 2011	0	1.5	50	4	3.7	50	26.5%	−1.41 [−1.85, −0.97]
22. Yuan 2016	14.25	2.32	30	15.59	1.52	30	22.9%	−0.67 [−1.20, −0.15]
31. Deng 2018	13.9	5.8	55	19.2	7.2	56	29.1%	−0.80 [−1.19, −0.42]
**Subtotal (95% CI)**			**160**			**163**	**100.0%**	**−0.89 [−1.25, −0.53]**
Heterogeneity: $Tau^2 = 0.08$; $Chi^2 = 7.14$, df = 3 (P = 0.07); $I^2 = 58\%$								
Test for overall effect: Z = 4.81 (P < 0.00001)								
**1.2.2 HFrEF***								
1. Barrow 2007	51.5	9.5	25	57.3	9.5	27	23.7%	−0.60 [−1.16, −0.04]
22. Yuan 2016	14.25	2.32	30	15.59	1.52	30	27.1%	−0.67 [−1.20, −0.15]
31. Deng 2018	13.9	5.8	55	19.2	7.2	56	49.1%	−0.80 [−1.19, −0.42]
**Subtotal (95% CI)**			**110**			**113**	**100.0%**	**−0.72 [−0.99, −0.45]**
Heterogeneity: $Tau^2 = 0.00$; $Chi^2 = 0.39$, df = 2 (P = 0.82); $I^2 = 0\%$								
Test for overall effect: Z = 5.20 (P < 0.00001)								
**1.2.3 HFpEF**								
20. Yeh 2013	2.3	2	8	3	3	8	100.0%	−0.26 [−1.25, 0.73]
**Subtotal (95% CI)**			**8**			**8**	**100.0%**	**−0.26 [−1.25, 0.73]**
Heterogeneity: Not applicable								
Test for overall effect: Z = 0.52 (P = 0.61)								
**1.2.4 HFrEF&HFpEF**								
34 Redwine 2019	6.1	6	18	7.73	6	33	34.0%	−0.27 [−0.84, 0.31]
35. Zheng 2018	6.11	3.15	47	7.07	3.15	44	66.0%	−0.30 [−0.72, 0.11]
**Subtotal (95% CI)**			**65**			**77**	**100.0%**	**−0.29 [−0.63, 0.05]**
Heterogeneity: $Tau^2 = 0.00$; $Chi^2 = 0.01$, df = 1 (P = 0.92); $I^2 = 0\%$								
Test for overall effect: Z = 1.69 (P = 0.09)								

Test for subgroup differences: $Chi^2 = 6.87$, df = 3 (P = 0.08), $I^2 = 56.3\%$

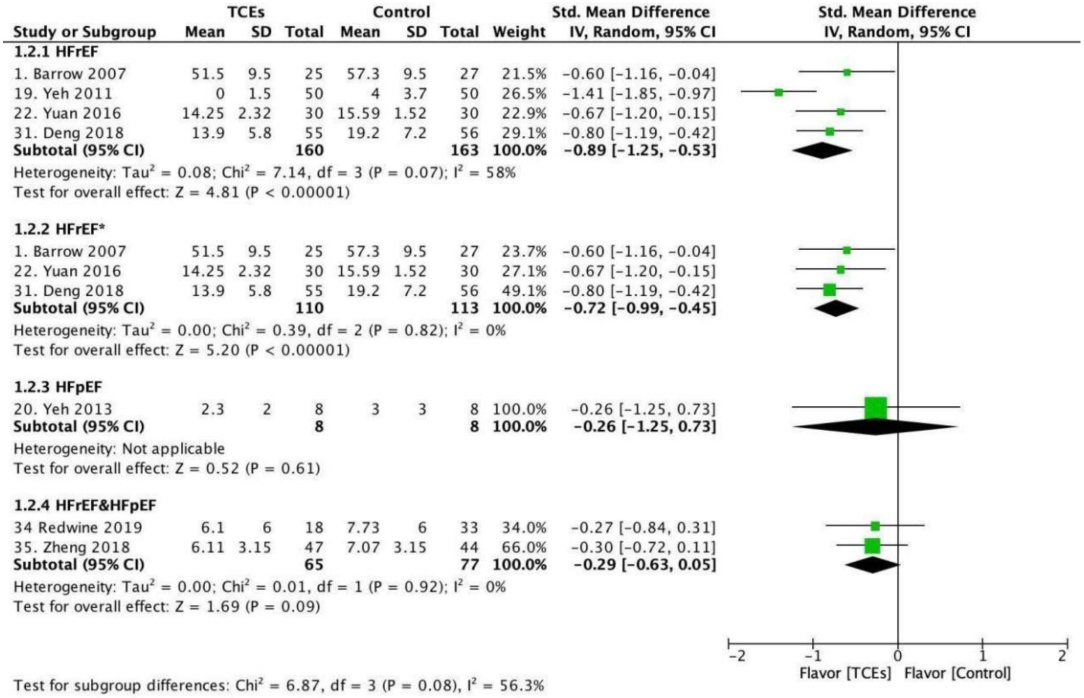

Subgroup analysis (Depressive severity)

Study or Subgroup	TCEs Mean	TCEs SD	TCEs Total	Control Mean	Control SD	Control Total	Weight	Std. Mean Difference IV, Random, 95% CI
**1.3.1 Mild**								
1. Barrow 2007	51.5	9.5	25	57.3	9.5	27	26.7%	−0.60 [−1.16, −0.04]
34 Redwine 2019	6.1	6	18	7.73	6	33	24.9%	−0.27 [−0.84, 0.31]
35. Zheng 2018	6.11	3.15	47	7.07	3.15	44	48.4%	−0.30 [−0.72, 0.11]
**Subtotal (95% CI)**			**90**			**104**	**100.0%**	**−0.37 [−0.66, −0.09]**
Heterogeneity: $Tau^2 = 0.00$; $Chi^2 = 0.89$, df = 2 (P = 0.64); $I^2 = 0\%$								
Test for overall effect: Z = 2.54 (P = 0.01)								
**1.3.2 Moderate**								
22. Yuan 2016	14.25	2.32	30	15.59	1.52	30	35.6%	−0.67 [−1.20, −0.15]
31. Deng 2018	13.9	5.8	55	19.2	7.2	56	64.4%	−0.80 [−1.19, −0.42]
**Subtotal (95% CI)**			**85**			**86**	**100.0%**	**−0.76 [−1.07, −0.45]**
Heterogeneity: $Tau^2 = 0.00$; $Chi^2 = 0.15$, df = 1 (P = 0.69); $I^2 = 0\%$								
Test for overall effect: Z = 4.78 (P < 0.00001)								
**1.3.3 Unclear**								
19. Yeh 2011	0	1.5	50	4	3.7	50	57.7%	−1.41 [−1.85, −0.97]
20. Yeh 2013	2.3	2	8	3	3	8	42.3%	−0.26 [−1.25, 0.73]
**Subtotal (95% CI)**			**58**			**58**	**100.0%**	**−0.92 [−2.03, 0.19]**
Heterogeneity: $Tau^2 = 0.51$; $Chi^2 = 4.34$, df = 1 (P = 0.04); $I^2 = 77\%$								
Test for overall effect: Z = 1.63 (P = 0.10)								
**1.3.4 Unclear***								
20. Yeh 2013	2.3	2	8	3	3	8	100.0%	−0.26 [−1.25, 0.73]
**Subtotal (95% CI)**			**8**			**8**	**100.0%**	**−0.26 [−1.25, 0.73]**
Heterogeneity: Not applicable								
Test for overall effect: Z = 0.52 (P = 0.61)								

Test for subgroup differences: $Chi^2 = 3.93$, df = 3 (P = 0.27), $I^2 = 23.7\%$

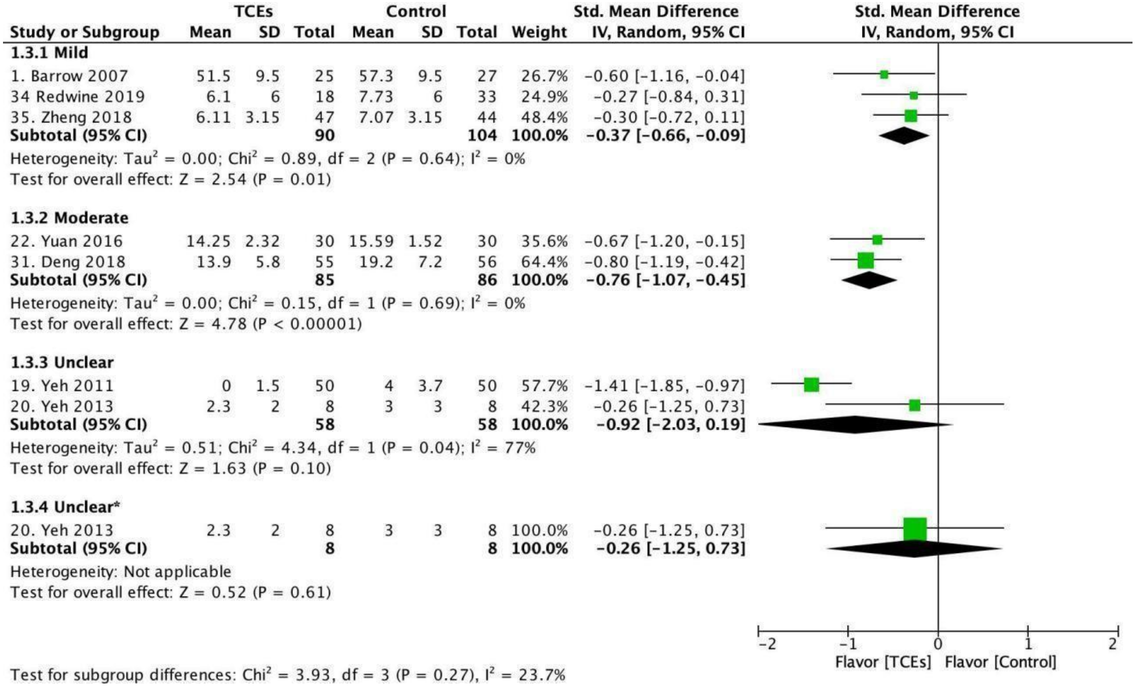

Subgroup analysis (Depression instrument)

Study or Subgroup	TCEs Mean	TCEs SD	TCEs Total	Control Mean	Control SD	Control Total	Weight	Std. Mean Difference IV, Random, 95% CI
**1.4.1 Clinician**								
22. Yuan 2016	14.25	2.32	30	15.59	1.52	30	35.6%	−0.67 [−1.20, −0.15]
31. Deng 2018	13.9	5.8	55	19.2	7.2	56	64.4%	−0.80 [−1.19, −0.42]
**Subtotal (95% CI)**			**85**			**86**	**100.0%**	**−0.76 [−1.07, −0.45]**
Heterogeneity: $Tau^2 = 0.00$; $Chi^2 = 0.15$, df = 1 (P = 0.69); $I^2 = 0\%$								
Test for overall effect: Z = 4.78 (P < 0.00001)								
**1.4.2 PRO/Specific**								
34 Redwine 2019	6.1	6	18	7.73	6	33	34.0%	−0.27 [−0.84, 0.31]
35. Zheng 2018	6.11	3.15	47	7.07	3.15	44	66.0%	−0.30 [−0.72, 0.11]
**Subtotal (95% CI)**			**65**			**77**	**100.0%**	**−0.29 [−0.63, 0.05]**
Heterogeneity: $Tau^2 = 0.00$; $Chi^2 = 0.01$, df = 1 (P = 0.92); $I^2 = 0\%$								
Test for overall effect: Z = 1.69 (P = 0.09)								
**1.4.3 PRO/Non-specific**								
1. Barrow 2007	51.5	9.5	25	57.3	9.5	27	36.2%	−0.60 [−1.16, −0.04]
19. Yeh 2011	0	1.5	50	4	3.7	50	39.7%	−1.41 [−1.85, −0.97]
20. Yeh 2013	2.3	2	8	3	3	8	24.1%	−0.26 [−1.25, 0.73]
**Subtotal (95% CI)**			**83**			**85**	**100.0%**	**−0.84 [−1.53, −0.15]**
Heterogeneity: $Tau^2 = 0.26$; $Chi^2 = 7.41$, df = 2 (P = 0.02); $I^2 = 73\%$								
Test for overall effect: Z = 2.38 (P = 0.02)								
**1.4.4 PRO/Non-specific***								
1. Barrow 2007	51.5	9.5	25	57.3	9.5	27	75.8%	−0.60 [−1.16, −0.04]
20. Yeh 2013	2.3	2	8	3	3	8	24.2%	−0.26 [−1.25, 0.73]
**Subtotal (95% CI)**			**33**			**35**	**100.0%**	**−0.52 [−1.00, −0.03]**
Heterogeneity: $Tau^2 = 0.00$; $Chi^2 = 0.35$, df = 1 (P = 0.55); $I^2 = 0\%$								
Test for overall effect: Z = 2.10 (P = 0.04)								

Test for subgroup differences: $Chi^2 = 4.68$, df = 3 (P = 0.20), $I^2 = 35.9\%$

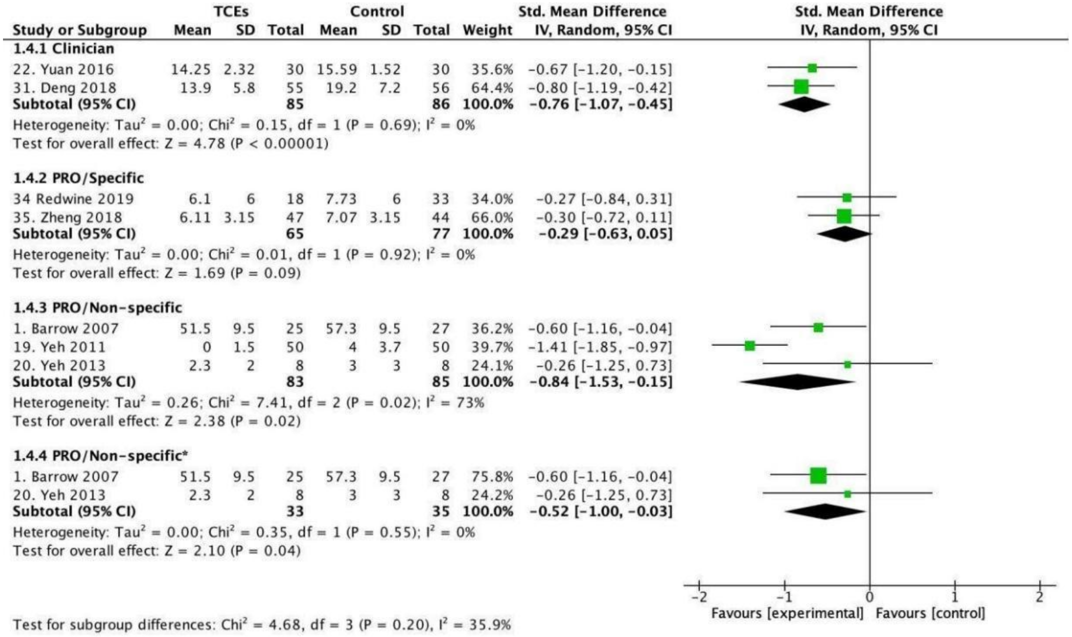

Subgroup analysis (TQPs)

Study or Subgroup	TCEs Mean	TCEs SD	TCEs Total	Control Mean	Control SD	Control Total	Weight	Std. Mean Difference IV, Random, 95% CI
**1.5.1 TaiChi**								
19. Yeh 2011	0	1.5	50	4	3.7	50	23.3%	−1.41 [−1.85, −0.97]
20. Yeh 2013	2.3	2	8	3	3	8	11.3%	−0.26 [−1.25, 0.73]
22. Yuan 2016	14.25	2.32	30	15.59	1.52	30	21.1%	−0.67 [−1.20, −0.15]
31. Deng 2018	13.9	5.8	55	19.2	7.2	56	24.8%	−0.80 [−1.19, −0.42]
34 Redwine 2019	6.1	6	18	7.73	6	33	19.6%	−0.27 [−0.84, 0.31]
**Subtotal (95% CI)**			**161**			**177**	**100.0%**	**−0.75 [−1.16, −0.34]**
Heterogeneity: $Tau^2 = 0.14$; $Chi^2 = 11.90$, df = 4 (P = 0.02); $I^2 = 66\%$								
Test for overall effect: Z = 3.57 (P = 0.0004)								
**1.5.2 TaiChi***								
20. Yeh 2013	2.3	2	8	3	3	8	7.2%	−0.26 [−1.25, 0.73]
22. Yuan 2016	14.25	2.32	30	15.59	1.52	30	25.6%	−0.67 [−1.20, −0.15]
31. Deng 2018	13.9	5.8	55	19.2	7.2	56	46.3%	−0.80 [−1.19, −0.42]
34 Redwine 2019	6.1	6	18	7.73	6	33	20.9%	−0.27 [−0.84, 0.31]
**Subtotal (95% CI)**			**111**			**127**	**100.0%**	**−0.62 [−0.88, −0.36]**
Heterogeneity: $Tau^2 = 0.00$; $Chi^2 = 2.86$, df = 3 (P = 0.41); $I^2 = 0\%$								
Test for overall effect: Z = 4.61 (P < 0.00001)								
**1.5.3 Qigong**								
35. Zheng 2018	6.11	3.15	47	7.07	3.15	44	100.0%	−0.30 [−0.72, 0.11]
**Subtotal (95% CI)**			**47**			**44**	**100.0%**	**−0.30 [−0.72, 0.11]**
Heterogeneity: Not applicable								
Test for overall effect: Z = 1.43 (P = 0.15)								
**1.5.4 TaiChi & Qigong**								
1. Barrow 2007	51.5	9.5	25	57.3	9.5	27	100.0%	−0.60 [−1.16, −0.04]
**Subtotal (95% CI)**			**25**			**27**	**100.0%**	**−0.60 [−1.16, −0.04]**
Heterogeneity: Not applicable								
Test for overall effect: Z = 2.12 (P = 0.03)								

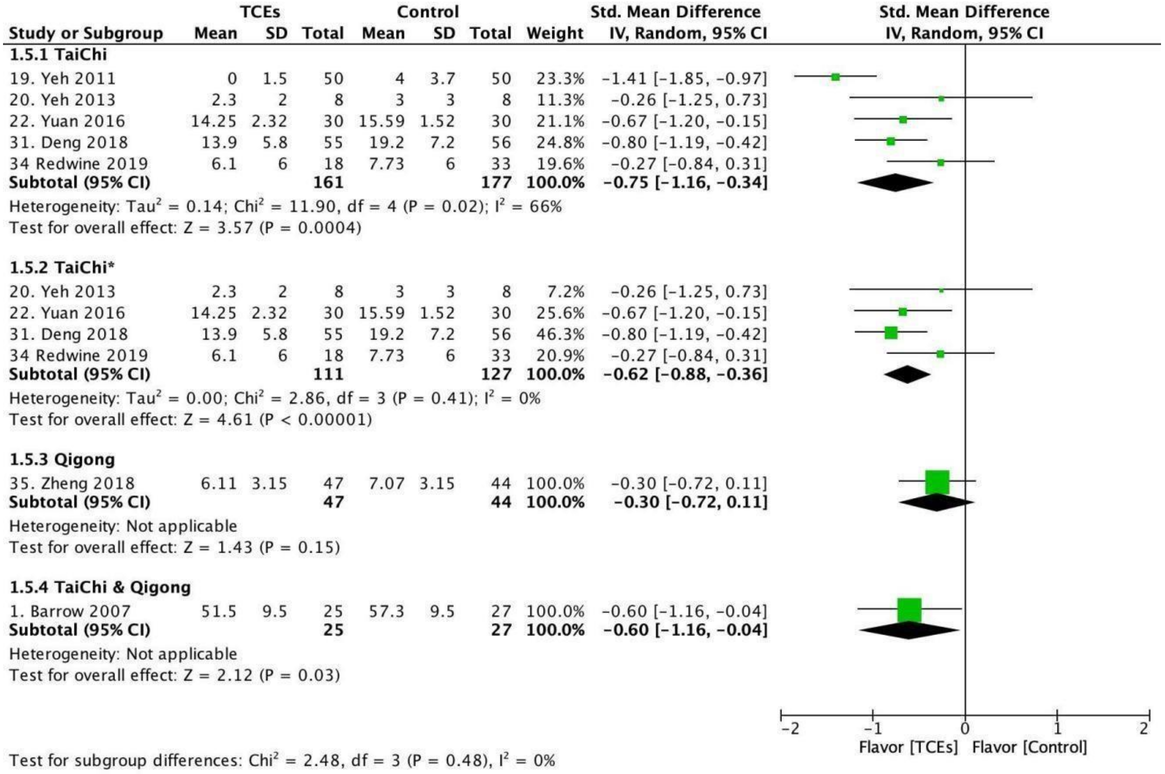

Test for subgroup differences: $Chi^2 = 2.48$, df = 3 (P = 0.48), $I^2 = 0\%$

Subgroup analysis (Length of TQP programs)

Study or Subgroup	TCEs Mean	TCEs SD	TCEs Total	Control Mean	Control SD	Control Total	Weight	Std. Mean Difference IV, Random, 95% CI
**1.6.1 12 weeks**								
19. Yeh 2011	0	1.5	50	4	3.7	50	28.0%	−1.41 [−1.85, −0.97]
20. Yeh 2013	2.3	2	8	3	3	8	17.1%	−0.26 [−1.25, 0.73]
22. Yuan 2016	14.25	2.32	30	15.59	1.52	30	26.3%	−0.67 [−1.20, −0.15]
35. Zheng 2018	6.11	3.15	47	7.07	3.15	44	28.6%	−0.30 [−0.72, 0.11]
**Subtotal (95% CI)**			**135**			**132**	**100.0%**	**−0.70 [−1.28, −0.12]**
Heterogeneity: $Tau^2 = 0.26$; $Chi^2 = 14.09$, df = 3 (P = 0.003); $I^2 = 79\%$								
Test for overall effect: Z = 2.37 (P = 0.02)								
**1.6.2 12 weeks***								
20. Yeh 2013	2.3	2	8	3	3	8	9.8%	−0.26 [−1.25, 0.73]
22. Yuan 2016	14.25	2.32	30	15.59	1.52	30	34.9%	−0.67 [−1.20, −0.15]
35. Zheng 2018	6.11	3.15	47	7.07	3.15	44	55.4%	−0.30 [−0.72, 0.11]
**Subtotal (95% CI)**			**85**			**82**	**100.0%**	**−0.43 [−0.74, −0.12]**
Heterogeneity: $Tau^2 = 0.00$; $Chi^2 = 1.33$, df = 2 (P = 0.52); $I^2 = 0\%$								
Test for overall effect: Z = 2.72 (P = 0.006)								
**1.6.3 >12 weeks**								
1. Barrow 2007	51.5	9.5	25	57.3	9.5	27	26.4%	−0.60 [−1.16, −0.04]
31. Deng 2018	13.9	5.8	55	19.2	7.2	56	48.8%	−0.80 [−1.19, −0.42]
34 Redwine 2019	6.1	6	18	7.73	6	33	24.8%	−0.27 [−0.84, 0.31]
**Subtotal (95% CI)**			**98**			**116**	**100.0%**	**−0.62 [−0.92, −0.31]**
Heterogeneity: $Tau^2 = 0.01$; $Chi^2 = 2.31$, df = 2 (P = 0.32); $I^2 = 13\%$								
Test for overall effect: Z = 3.99 (P < 0.0001)								

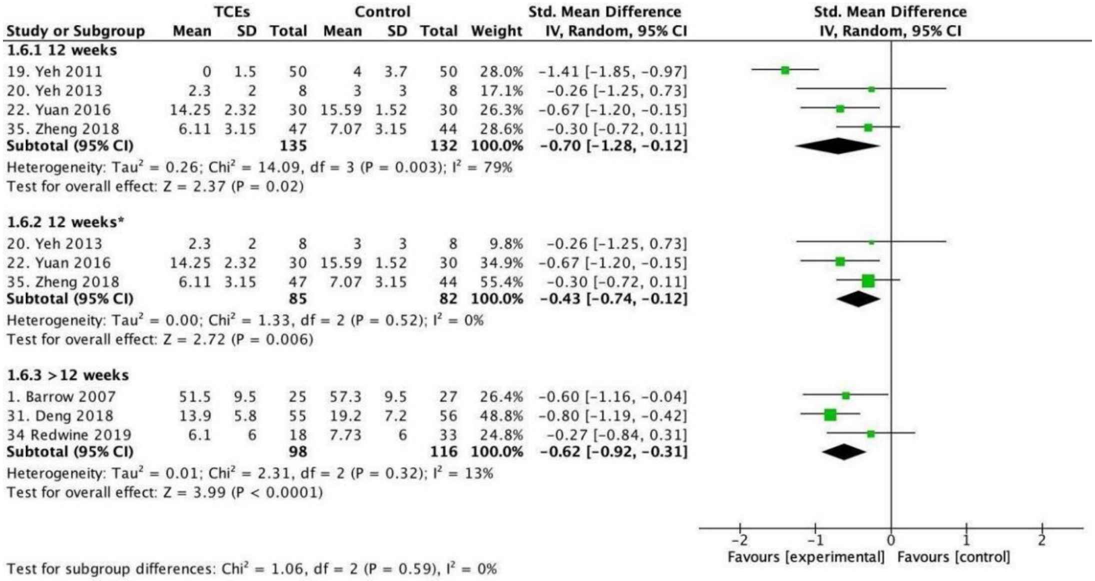

Test for subgroup differences: $Chi^2 = 1.06$, df = 2 (P = 0.59), $I^2 = 0\%$

Subgroup analysis (TQP delivery settings)

	TCEs			Control				Std. Mean Difference
Study or Subgroup	Mean	SD	Total	Mean	SD	Total	Weight	IV, Random, 95% CI
**1.7.1 Center-based/Outside China**								
1. Barrow 2007	51.5	9.5	25	57.3	9.5	27	26.6%	−0.60 [−1.16, −0.04]
19. Yeh 2011	0	1.5	50	4	3.7	50	29.1%	−1.41 [−1.85, −0.97]
20. Yeh 2013	2.3	2	8	3	3	8	18.0%	−0.26 [−1.25, 0.73]
34 Redwine 2019	6.1	6	18	7.73	6	33	26.2%	−0.27 [−0.84, 0.31]
**Subtotal (95% CI)**			**101**			**118**	**100.0%**	**−0.69 [−1.29, −0.08]**
Heterogeneity: Tau² = 0.28; Chi² = 12.18, df = 3 (P = 0.007); I² = 75%								
Test for overall effect: Z = 2.22 (P = 0.03)								
**1.7.2 Center-based/Outside China***								
1. Barrow 2007	51.5	9.5	25	57.3	9.5	27	44.4%	−0.60 [−1.16, −0.04]
20. Yeh 2013	2.3	2	8	3	3	8	14.2%	−0.26 [−1.25, 0.73]
34 Redwine 2019	6.1	6	18	7.73	6	33	41.4%	−0.27 [−0.84, 0.31]
**Subtotal (95% CI)**			**51**			**68**	**100.0%**	**−0.41 [−0.79, −0.04]**
Heterogeneity: Tau² = 0.00; Chi² = 0.78, df = 2 (P = 0.68); I² = 0%								
Test for overall effect: Z = 2.19 (P = 0.03)								
**1.7.3 Home-based**								
22. Yuan 2016	14.25	2.32	30	15.59	1.52	30	26.1%	−0.67 [−1.20, −0.15]
31. Deng 2018	13.9	5.8	55	19.2	7.2	56	38.4%	−0.80 [−1.19, −0.42]
35. Zheng 2018	6.11	3.15	47	7.07	3.15	44	35.5%	−0.30 [−0.72, 0.11]
**Subtotal (95% CI)**			**132**			**130**	**100.0%**	**−0.59 [−0.91, −0.28]**
Heterogeneity: Tau² = 0.03; Chi² = 3.14, df = 2 (P = 0.21); I² = 36%								
Test for overall effect: Z = 3.69 (P = 0.0002)								

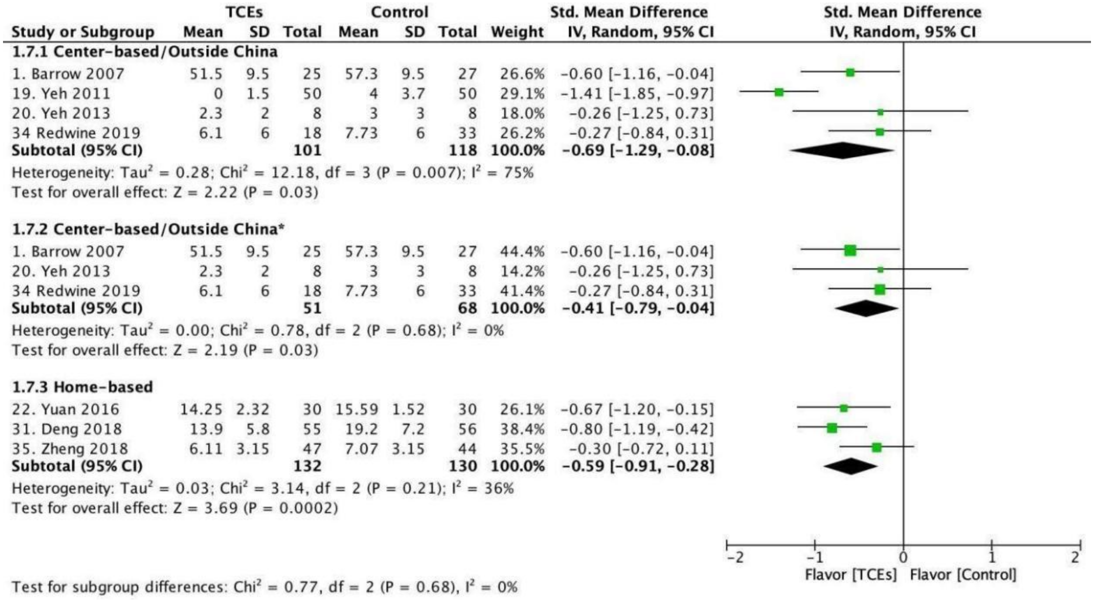

Test for subgroup differences: Chi² = 0.77, df = 2 (P = 0.68), I² = 0%

Appendix 3. Risk-of-bias analysis.

(A) Risk of bias summary: review author judgments about each risk of bias item for each includedstudy
(B) Risk-of-bias graph: The authors' judgments regarding each risk-of-bias item, presented as percentages across all included studies

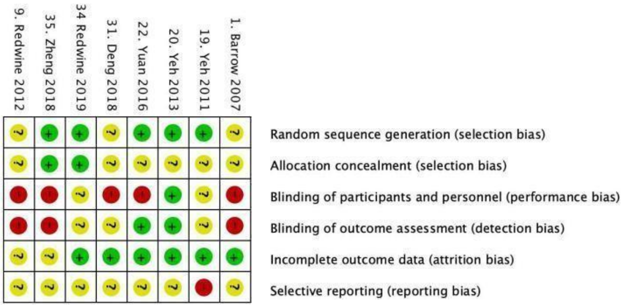

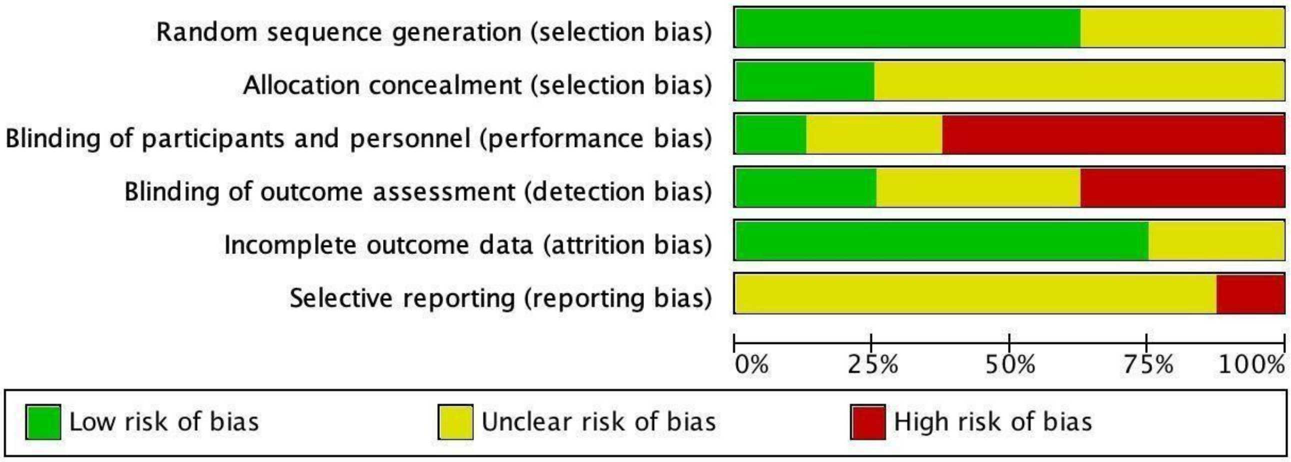

Supplement: Supplementary Materials — Appendix 1: search strategy. Appendix 2: subgroup analysis. Appendix 3: risk of bias analysis. Appendix 4: PRISMA 2009 checklist. [file 5585239.f1.zip › 5585239.f1/5585239_1stRevision_2 Appendix 1-3_SR for CHF on depression.pdf]
